# WHO urges more effective prevention of injuries and violence causing 1 in 12 deaths worldwide

**Published:** 2022-12

**29 November 2022** - WInjuries and violence take the lives of some 12 000 people around the world each day. As reflected in a new World Health Organization report, Preventing injuries and violence: an overview, 3 of the top 5 causes of death among people aged 5–29 years are injury related, namely road traffic injuries, homicide and suicide.

In addition to those, injury related killers are drowning, falls, burns and poisoning, among others. Of the 4.4 million annual injury related deaths, roughly 1 in 3 of these deaths result from road traffic crashes, 1 in 6 from suicide, 1 in 9 from homicide and 1 in 61 from war and conflict.

“People living in poverty are significantly more likely to suffer an injury than the wealthy,” said Dr Tedros Adhanom Ghebreyesus, WHO Director-General. “The health sector has a major role in addressing these health inequities and in preventing injuries and violence, through collecting data, developing policies, providing services and programming for prevention and care, building capacities, and advocating for greater attention to underserved communities.”

Many effective and low-cost interventions are available. For example, in Spain, setting the default speed limit for cities at 30 kilometres per hour is improving road safety; in Viet Nam, providing swimming training is preventing drowning; and in the Philippines, legislation to raise the age of sexual consent from 12 years to 16, in a bid to protect minors from sexual violence, is bringing positive change. However, in most countries, political will and investment are lacking as measures are not in place in sufficient levels.

“Accelerated action is needed to avoid this unnecessary suffering of millions of families every year,” notes Dr Etienne Krug, Director of the Department for the Social Determinants of Health, WHO. “We know what needs to be done, and these effective measures must be brought to scale across countries and communities to save lives.”

The WHO report is being released during the 14th World Conference on Injury Prevention and Safety Promotion, currently taking place in Adelaide, Australia. This event provides an opportunity for the world’s leading injury and violence prevention researchers and practitioners to continue to advocate for evidence-based measures to prevent injuries and violence.

This report also highlighted the prevention measures and available WHO technical guidance that can support decisions for scaling up prevention efforts.

Available from: **https://www.who.int/news/item/29-11-2022-who-urges-more-effective-prevention-of-injuries-and-violence--causing-1-in-12-deaths-worldwide**

